# Great auricular nerve schwannoma in neck region: a case report with the risk of medical disputes

**DOI:** 10.1186/s12883-019-1532-y

**Published:** 2019-12-01

**Authors:** Chunling Xu, Qingjia Sun

**Affiliations:** 1 Department of Ophthalmology, The Second Hospital of Jilin University, Nanguan, Changchun, Jilin, 130041 People’s Republic of China; 2 Department of Otorhinolaryngology Head and Neck Surgery, The China-Japan Union Hospital of Jilin University, Xiantai Street 126, Changchun, 130033 China

**Keywords:** Schwannoma, Great auricular nerve, Medical disputes

## Abstract

**Background:**

Great auricular nerve schwannoma is extremely rare. Herein, we reported the first case of schwannoma arising from great auricular nerve trunk.

**Case presentation:**

A 29 year-old female complained of a slowly-growing superfacial neck mass for 6 months. MRI revealed a high possibility of schwannoma. Although the patient underwent successfully surgical removal of the tumor, ipsilateral numbness of both auricle and peripheral skin developed due to traction of the nerve. Immunohistochemistry staining confirmed the diagnosis of schwannoma. And the patient has been followed regularly.

**Conclusion:**

For superficial cervical tumors, the cervical plexus cutaneous nerve should be considered if MRI and other imaging findings suggest neurogenic tumors.

## Background

Schwannoma is a benign neurogenic tumor originated from Schwann cells within peripheral nerve sheath. The disease commonly occurs in the head and neck region with an incidence of 25–45% [1]. Schwannoma in the head and neck region mostly originate from the eighth cranial nerve (vestibulocochlear nerve) [2], and it was most common in parapharyngeal space for the neck region. Schwannoma may also occur on other sites including the face, scalp, parotid gland (facial nerves), oral cavity, pharynx, larynx, and trachea [3]. In this case report, we presented a schwannoma originated from the great auricular nerve in the neck region.

## Case presentation

A 29 year-old female was referred to the department of otorhinolaryngology head and neck surgery of the China–Japan union hospital of Jilin University, with a complaint of a slowly-growing mass in the neck region.

The physical examination showed that the mass was located approximately 2 cm below the lower margin of left mandible and adjacent to the anterior margin of sternocleidomastoid muscle. The mass was tough and unfixed without pressing pain.

**Fig. 1:**
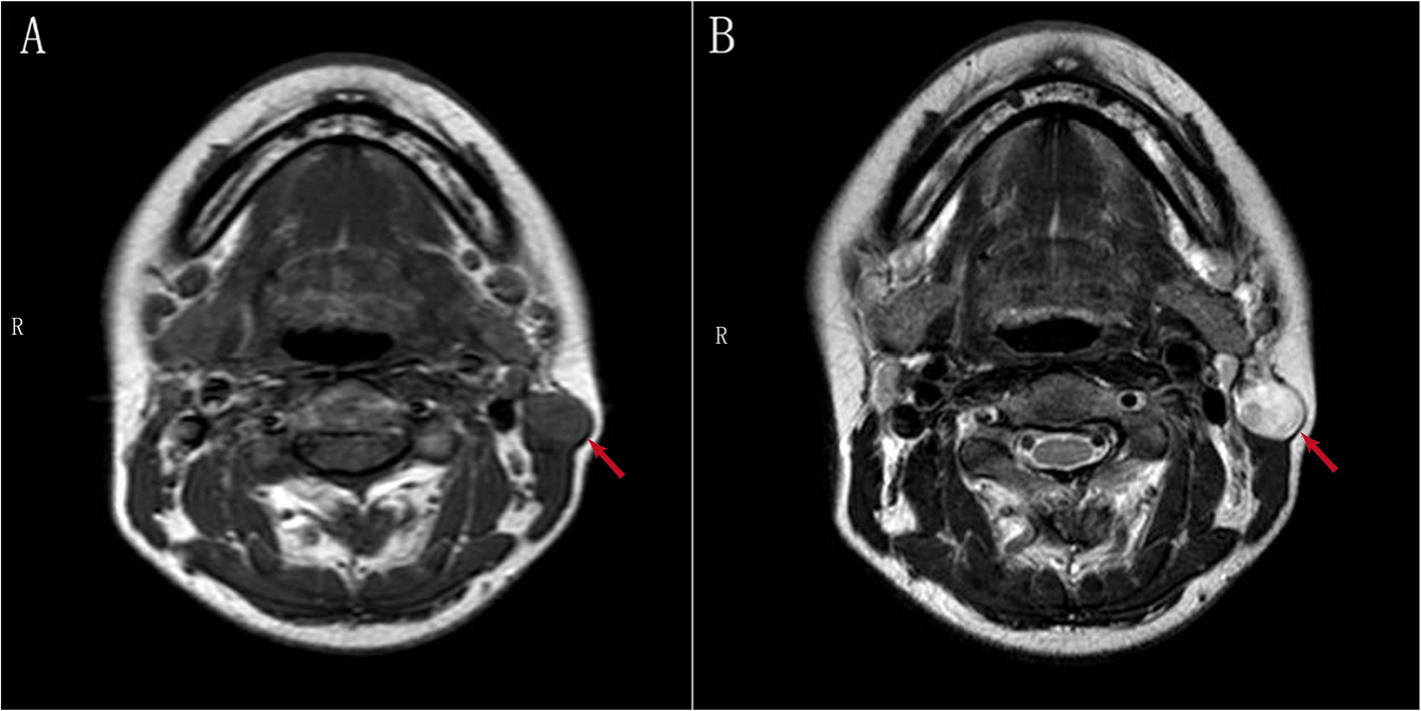
MRI revealed a high possibility of schwannoma:a subcutaneous nodule in left neck with intermediate T1 (**a**) and T2 signal (**b**) as well as clear margin (red arrow). The mass presented inhomogeneous intermediate signal at T2 similar with Antoni A as well as Antoni B area (**b**)

Magnetic resonance imaging (MRI) showed a subcutaneous nodule in left neck with intermediate signal at both T1 and T2. MRI revealed a well-defined round-like mass (diameter ~ 1.5 cm) located on the superficial surface of sternocleidomastoid muscle. The mass presented inhomogeneous intermediate signal at T2 similar with Antoni A as well as Antoni B area. All the information above suggested a high possibility of schwannoma (Fig. 1).

Based on the clinical examination and imaging information, a surgery was performed subsequently. Patient was informed about provisional diagnosis, treatment options and potential postoperative complications. And the written consent was obtained from the patient as well. The treatment plan was approved by the institutional review board and ethics committee of our hospital.

**Fig. 2:**
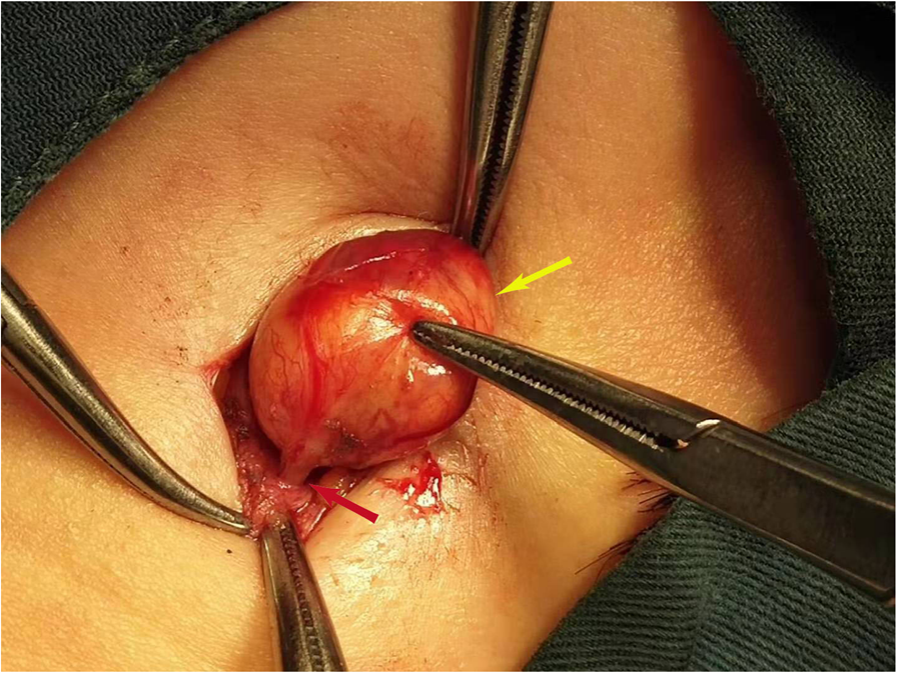
Tumor connected with the nerve: the round-like tumor (yellow arrow) is smooth, well-encapsulated and well-defined with surrounding tissues, the upper and lower poles of which are seen to be connected with a nerve (red arrow)

**Fig. 3:**
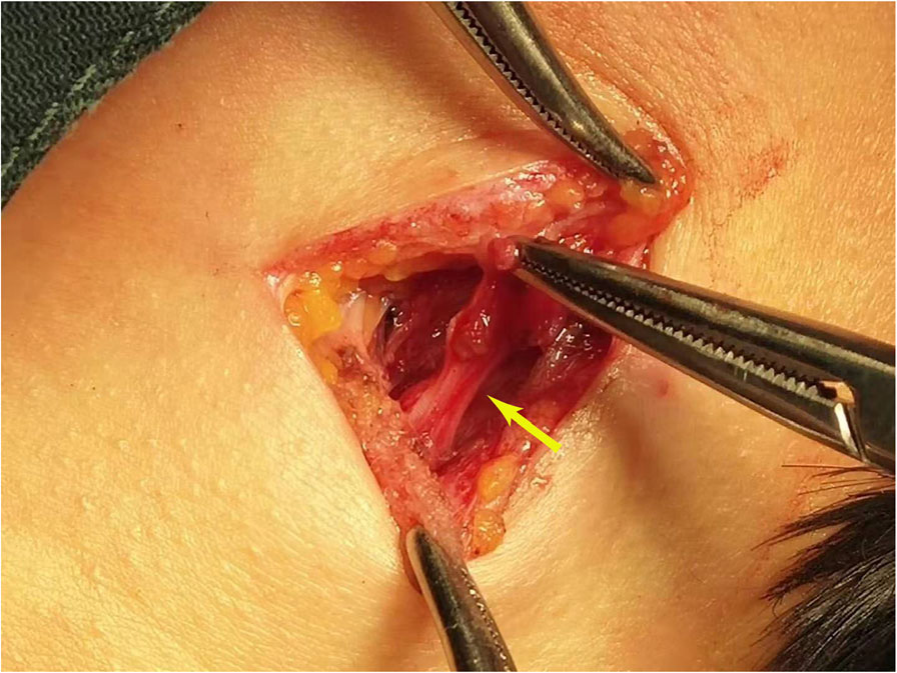
The mass was removed by sharp dissection with the integrity of the capsule (yellow arrow)

External resection was performed under local anesthesia through a small incision. During the operation, a round-like tumor was observed. The tumor was smooth, well-encapsulated and well-defined with surrounding tissues. The upper and lower poles of the mass adhered to the nerve (Fig. 2). The mass was removed by sharp dissection with the integrity of the capsule (Fig. 3). And the nerve was accompanied by external jugular vein.

Thereafter, the histopathological study described a benign cluster of spindle cells without mitosis, atypia or necrosis, but with some verocay bodies. The cells showed intense immunoreactivity for protein S-100. All characteristics were consistent with the diagnosis of schwannoma.

The patient developed immediate ipsilateral numbness of both auricle and peripheral skin due to traction of the nerve postoperatively. As to the superfacial mass of neck, we firstly took abnormal lymph node into account for that schwannoma originated from the trunk of the greater auricular nerve had not been reported previously. However, radiologists considered the possibility of schwannoma via MRI features. Therefore, we thought that the mass may originate from small sensory nerve fibers. Auricular numbness was not explained to the patient, nor was it reflected in the informed consent form before surgery. We explained to the patient about the causes of auricular numbness, and patient understood our explanation.

The local healing of the patient was good, and the suture was removed on the seventh day. Then the patient was followed-up regularly (every 3–6 months) [4].

## Discussion and conclusion

Great auricular nerve schwannoma is extremely rare. According to the literature review, it was the first case of great auricular nerve schwannoma in neck region. Adhikary B et al. reported a solitary schwannoma in the postaural region [5]. Schwannoma was not reported originating from the trunk of the greater auricular nerve before, so we ignored the potential complication of auricle numbness. Although no disputes happened in this case, we immediately shared the case to otolaryngologists in countries with high possibility of medical disputes and violence such as China, Israel and India [6–8].

In this case, three diagnostic evidences confirmed that the tumor originated from the great auricular nerve: 1) within operation, the direction of the nerve coincides with the great auricular nerve. 2) after tumor resection, regional numbness of great auricular nerve innervation occurred. 3) characteristics of the mass meets with the diagnostic criteria for Enzinger and Weiss histologically.

In the case report, surgery was performed. To the best of our knowledge, surgery was more reported as the first choice for extracranial head and neck schwannomas, possibly due to radio-resistance [1, 9, 10].. Unlike malignancies, postoperative complications for benign, small and superficial tumors may be not acceptable by patients. For superficial cervical tumors, the cervical plexus cutaneous nerve should be considered if MRI or other imaging materials suggest neurogenic tumors. In this case, the trunk damage caused by removal of a mass, resulted in the regional numbness.

## Data Availability

The datasets used and/or analysed during the current study available from the corresponding author on reasonable request.

## Abbreviation

MRI: Magnetic resonance imaging
